# Tackling Cancer Resistance by Immunotherapy: Updated Clinical Impact and Safety of PD-1/PD-L1 Inhibitors

**DOI:** 10.3390/cancers10020032

**Published:** 2018-01-25

**Authors:** Shifaa M. Abdin, Dana M. Zaher, El-Shaimaa A. Arafa, Hany A. Omar

**Affiliations:** 1 College of Pharmacy, University of Sharjah, Sharjah 27272, UAE; U00044748@sharjah.ac.ae; 2 Sharjah Institute for Medical Research, University of Sharjah, Sharjah 27272, UAE; U17105878@sharjah.ac.ae; 3 Department of Pharmacology and Toxicology, College of Pharmacy and Health Sciences, Ajman University, Ajman 346, UAE; e.arafa@ajman.ac.ae; 4 Department of Pharmacology and Toxicology, Faculty of Pharmacy, Beni-Suef University, Beni-Suef 62514, Egypt

**Keywords:** cancer, immunotherapy, PD-1, PD-L1, checkpoint inhibitors, nivolumab, pembrolizumab, pidilizumab, atezolizumab, durvalumab, avelumab

## Abstract

Cancer therapy has been constantly evolving with the hope of finding the most effective agents with the least toxic effects to eradicate tumors. Cancer immunotherapy is currently among the most promising options, fulfilling this hope in a wide range of tumors. Immunotherapy aims to activate immunity to fight cancer in a very specific and targeted manner; however, some abnormal immune reactions known as immune-related adverse events (IRAEs) might occur. Therefore, many researchers are aiming to define the most proper protocols for managing these complications without interfering with the anticancer effect. One of these targeted approaches is the inhibition of the interaction between the checkpoint protein, programmed death-receptor 1 (PD-1), and its ligand, programmed death-ligand 1 (PD-L1), via a class of antibodies known as PD-1/PD-L1 inhibitors. These antibodies achieved prodigious success in a wide range of malignancies, including those where optimal treatment is not yet fully identified. In this review, we have critically explored and discussed the outcome of the latest PD-1 and PD-L1 inhibitor studies in different malignancies compared to standard chemotherapeutic alternatives with a special focus on the clinical efficacy and safety. The approval of the clinical applications of nivolumab, pembrolizumab, atezolizumab, avelumab, and durvalumab in the last few years clearly highlights the hopeful future of PD-1/PD-L1 inhibitors for cancer patients. These promising results of PD-1/PD-L1 inhibitors have encouraged many ongoing preclinical and clinical trials to explore the extent of antitumor activity, clinical efficacy and safety as well as to extend their applications.

## 1. Introduction

Cancer therapy has regularly shown glimmers of hope in the search for the most effective measure with the least toxic effects to eradicate tumors. Various approaches have been implemented in cancer therapy, which include surgery, radiation, chemotherapy and targeted therapy. The continuous process of research has shifted the focus in the last century toward immunotherapy as a novel tool for fighting cancer. The concept of utilizing immunity as a therapeutic technique was first introduced in 1957 by Thomas and Burnett, who discussed the ability of tumors to provoke an immune response [1]. Then, it was verified that the human immune system can recognize and eliminate malignant cells through both the innate and adaptive systems [2,3]. Thus, cancer immunotherapy was established based on modulating the immune system to combat tumors in contrast to the traditional strategies that aim to target tumors for eradication [4,5].

The growing evidence on the role of immunity in treating cancer has given rise to multiple strategies such as the inhibition of cancer checkpoint proteins. Checkpoint proteins are considered a vital part of the immune system expressed on different immune cells with the ability to initiate an immune response either by their activation or inhibition [6]. In 1997, Rituximab was the first approved immunomodulatory monoclonal antibody (mAb) in cancer immunotherapy targeting the B-lymphocyte antigen, CD20, in non-Hodgkin lymphoma [7].

The activation of checkpoint proteins expressed on T-lymphocytes such as cytotoxic T-lymphocyte antigen 4 (CTLA-4) and PD-1, usually helps the tumor cells to escape detection by the immune system. Thus, the inhibition of these checkpoint proteins would activate the immune system against tumors [8]. Compared to chemotherapy and targeted therapy, checkpoint inhibitors have demonstrated higher rates of response, remission, and overall better survival rates in numerous kinds of tumors [9]. However, the primary documented cancer resistance with the use of PD-1 pathway inhibitors aroused initial doubts regarding their clinical applications [10]. In addition, IRAEs associated with the use of checkpoint inhibitors might hinder their application [11]. PD-1 receptors are related to the CD28 family, which is located on numerous immune cells, including T and B lymphocytes and natural killers. PD-L1 (B7-H1, CD274) is a protein that belongs to the B7 family, which acts as a ligand for PD-1 receptors. PD-L1 expression is diverse as it can be located on some of the tumor-infiltrating immune cells, including myeloid-derived suppressor cells, T cells, B cells, monocytes, macrophages and dendritic cells. In addition, the presence of PD-L1 was documented on different types of non-hematopoietic tissues [12]. Moreover, it can be upregulated in some types of cancers that utilize these checkpoints to escape immune defenses through the interaction between PD-L1 ligands expressed in these tumors and the inhibitory PD-1 receptors on immune cells [12].

**Figure 1:**
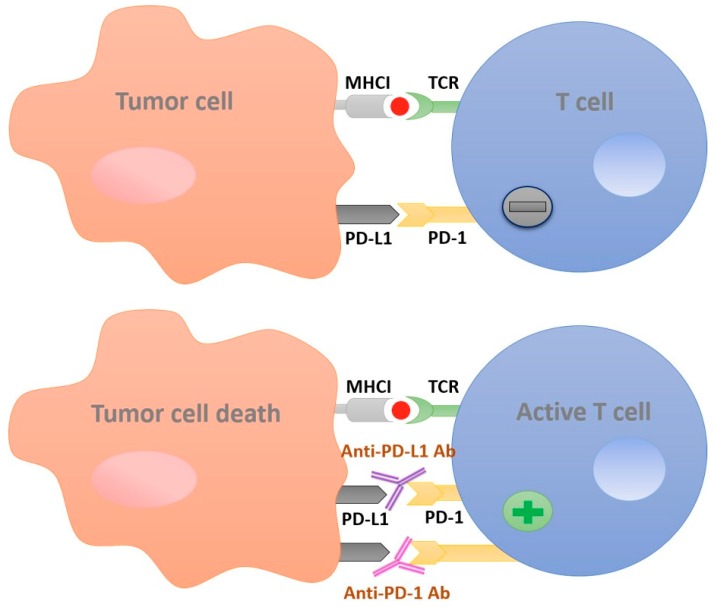
Diagram depicting the anticancer mechanism of PD-1/PD-L1 inhibitors. Tumor cells escape from the anti-tumor activity of T cells by the binding of PD-L1 to the PD-1 receptor. PD-1 or PD-L1 antibodies block the binding of PD-L1 on tumor cells to PD-1 receptors on T cells, which allows T cells to induce the immune response against tumor cells. MHCI, Major histocompatibility complex I; TCR, T cell receptor; PD-L1, Programmed death-ligand 1; PD-1, Programmed death-receptor 1; Ab, antibody.

This binding acts as an off-switch to immunity, preventing the activation of immune cells against cancer cells [13]. PD-1/PD-L1 inhibitors block this binding, and help the immune cells to regain their defensive role (Figure 1). However, in some types of malignancies, the expression of PD-L1 is induced by the inflammatory mediator, IFN-γ, from the tumor stromal cells which inhibits CD8^+^ cytotoxic T cells from inducing an anti-tumor response. Thus, tumors would acquire an adaptive resistance through PD-L1 upregulation, which is one of the obstacles against the application of PD-1/PD-L1 inhibitors [14].

The efficacy of PD-1/PD-L1 pathway inhibitors was demonstrated in over 15 types of tumors with high success rates compared to traditional chemotherapy [15]. One of these explored malignancies is the non-small cell lung cancer (NSCLC). Since lung cancer was not considered immunogenic, the association between immune response and lung cancer was discussed historically to be of poor strength [16]. However, counteracting evidence came to the fore in the discovery of the expression of PD-L1 in lung tumor cells, which suggested the role of T-cells in combatting NSCLC [17]. The efficacy of PD-L1 inhibitors was initially verified in different animal models of NSCLC before the approval of many PD-L1 inhibitors for NSCLC treatment [18,19].

Since these agents target the immune system, tumors whose growth and development are affected by the immune system would be greatly affected. For example, in metastatic melanoma (MM), many approaches targeting the immune system have been used and thus, immunotherapy for MM is well defined [20]. The U.S. food and drug administration (FDA) first approved the use of interferon and interleukin 2 (IL-2) for melanoma based on better responses compared to the use of classical chemotherapy [21]. The first immune checkpoint inhibitor approved for MM was ipilimumab, a CTLA-4 inhibitor [22]. Another immune checkpoint inhibitor, nivolumab, which is a PD-1 inhibitor was FDA approved for MM treatment [23]. The urothelial bladder cancer is another malignancy where multiple PD-1/PD-L1 inhibitors are FDA approved for the treatment. This tumor has a well-known immunogenicity, due to its marked high expression levels of immune-related factors such as PD-L1 and other immune markers [24]. Therefore, any cancer with proven expression of the PD-L1 ligand should be a potential target for these checkpoint inhibitors. 

In this review, we have critically explored and discussed the outcome of the latest PD-1 and PD-L1 inhibitor studies in different malignancies compared to standard chemotherapeutic alternatives with a special focus on the clinical efficacy and safety.

## 2. PD-1 Inhibitors

### 2.1. Nivolumab

Nivolumab (BMS-936558/MDX-1106/ONO 4538), an IgG-4 mAb, is a PD-1 inhibitor, which has a broad spectrum of activity in different types of tumors (Table 1) [23,25]. Many clinical studies have demonstrated the superiority of nivolumab over chemotherapy and provided evidence of tolerability when it comes to patients’ survival rates and response parameters [24]. In light of the continuous and successful results of nivolumab use in MM patients, in 2014, the FDA granted nivolumab accelerated approval for its use in MM patients who did not respond to other treatments [26]. One of the phase III trials compared the efficacy of nivolumab to dacarbazine, a chemotherapeutic agent, as a first-line treatment in MM patients with negative B-RAF mutation [27]. In this study, 418 MM patients were randomized to receive either dacarbazine or nivolumab at a dose of 300 mg/kg every two weeks. The 1-year survival rate of patients on nivolumab was 73%, and only 11.7% of them displayed adverse events. On the other hand, 42% of patients who received chemotherapy (dacarbazine) had an overall survival (OS) rate of one year and 17.6% displayed adverse events [27]. Thus, the conducted trial confirmed the superiority of nivolumab over chemotherapy due to a higher response rate and better tolerability. In another phase I/II cohort study, a total of 107 melanoma pretreated patients were enrolled, to receive nivolumab intravenously twice a week at a dose range of 0.1–10 mg/kg. The median OS was 17 months and the objective response rate (ORR) was 31% [23].

Other studies were performed to explore the effect of nivolumab in NSCLC treatment. Some of these trials assessed its efficacy as a second-line treatment upon the initiation of other types of therapy. The latest related preformed trials are the CheckMate 017 and CheckMate 57 [28,29]. Both are phase III trials, which were performed on patients with advanced NSCLC after or during the initiation of platinum-based drugs as chemotherapy. These trials randomized the patients into two groups, either to receive docetaxel or nivolumab and both trials showed results favoring nivolumab over docetaxel. The CheckMate 017 trial, which was implemented on 272 patients, resulted in a median OS with nivolumab of 9.2 months compared to 6.0 months with docetaxel, and a higher ORR with nivolumab (20%) compared to (9%) within docetaxel-treated patients. This trial has also demonstrated a higher safety profile for nivolumab, in which only 7% of the nivolumab-treated group displayed grade 3–4 therapy-related adverse events, unlike the docetaxel group, where the adverse events were exhibited in 55% of treated patients [28]. In addition, CheckMate 57 confirmed the previous results, but on a larger scale, as it was applied on 582 patients with the same conditions as CheckMate 017. CheckMate 57 showed the superiority of nivolumab with the following results: median OS with nivolumab recorded as 12.2 months (95% CI, 9.7–15.0), while in the docetaxel arm, the median OS was recorded as 9.4 months (95% CI, 8.0–10.7) [29].

These findings were the reasons behind the FDA approval of nivolumab use as a second-line treatment in advanced NSCLC, in 2015 [28]. Further trials to investigate nivolumab efficacy as a sole first-line agent in NSCLC were performed. The CheckMate 012 trial showed an ORR of 28% in NSCLC patients with a positive PD-L1 expression in patients treated with nivolumab [30]. However, nivolumab showed no superiority when compared to platinum-based chemotherapy as a first-line agent, according to the CheckMate 026 trial results [48]. These studies concluded that enough evidence has been collected, making the use of nivolumab as a second-line treatment in both MM and NSCLC highly recommended. Nonetheless, there is no doubt that nivolumab would be of great benefit in MM, since it is known to be one of the most immunogenic cancers [49]. Currently, many ongoing trials are exploiting the use of nivolumab in other types of solid tumors such as gastric cancer. A phase III trial was performed to investigate the capacity of nivolumab in solving the poor prognosis of gastroesophageal junction cancer (GEJC). In this trial, a group of 493 patients, who failed to achieve a response to chemotherapy, were randomized to receive nivolumab or a placebo. Patients treated with nivolumab showed superior results to the placebo with a median OS of 5.32 months vs. 4.14 months with the placebo and an ORR of 11.2% compared to 0% with the placebo. Based on these findings, nivolumab would be a favored treatment option for GEJC [31]. Furthermore, CheckMate 032, a phase I/II study, was preformed specifically on advanced and metastatic cases of GEJC. This study presented supporting evidence of the previously demonstrated tolerance and response data upon the use of nivolumab as a monotherapy in this type of gastric cancer. Among the 59 enrolled PD-L1-positive patients, 18% were noted as an ORR [32]. Nivolumab has again proved its superiority over the regular use of chemotherapy in Head and Neck cancer, according to the randomized phase III trial. The CheckMate 141 study enrolled 361 Head and Neck cancer patients, all of whom were previously treated with platinum-based compounds. In this study, patients were given either nivolumab at a dose of 3 mg/kg every two weeks or the researcher’s selection of chemotherapeutic drug. The nivolumab group achieved 7.5 months median OS compared to 5.1 months with the chemotherapy group; in addition, nivolumab-treated patients had lower death risk by 30% [25]. Lastly, nivolumab was also investigated in ovarian cancer; two cohort studies were conducted in which a total of 20 chemotherapy-resistant patients were treated with nivolumab at a 1 mg/kg or 3 mg/kg dose. Among the 20 enrolled patients, the overall median survival was 20 months; the best overall response was 15% and only eight patients showed grade 3–4 adverse events [33]. All the previously described uses of nivolumab across different malignancies share a higher clinical efficacy and a lower potential for treatment-related adverse events compared to chemotherapy. However, in rare cases, nivolumab can result in potentially more harmful side effects than displayed by chemotherapy-treated patients, as shown by IRAEs. One of the life threatening IRAEs is interstitial lung disease (ILD), which was reported to be an adverse event resulting from nivolumab use in three NSLC patients; two out of those three patients died of respiratory failure [50]. This contradicts with the encouraging results that favored nivolumab use over chemotherapy in NSCLC patients. To conclude, the IRAEs associated with nivolumab represent a drawback, especially when it comes to nivolumab recognition as a first-line agent. Hence, further investigation is required to target the prevalence of such deadly side effects, and to define the type of tumor in which nivolumab application would be the best option.

### 2.2. Pembrolizumab

Pembrolizumab (MK-3475) is an IgG-1 mAb that is considered to be a highly potent and selective PD-1 inhibitor [51]; it was approved by the FDA for the treatment of both MM and NSCLC (Table 1) [52,53]. In NSCLC, pembrolizumab displayed a great efficacy as a first- and second-line treatment. The KEYNOTE-001 phase II/III trial is one of the studies that confirmed pembrolizumab’s success in NSCLC. This trial enrolled 1034 NSCLC patients previously treated with chemotherapy, immunotherapy, epidermal growth factor receptor (EGFR) inhibitor or anaplastic lymphoma kinase (ALK) inhibitor who had positive PD-L1 expression. Patients in this trial were randomized to receive either 2 mg/kg or 10 mg/kg of pembrolizumab in addition to a group of patients who received 75 mg/m² of docetaxel, administered every 3 weeks. Parallel to nivolumab trials, the results were in favor of pembrolizumab over docetaxel as a chemotherapeutic agent in the two different regimens. The median OS was 10.4 and 12.7 months for those who received 2 mg/kg and 10 mg/kg of pembrolizumab, respectively. On the other hand, docetaxel patients had 8.5 months as a median OS. Furthermore, better tolerance was noted in patients treated with pembrolizumab, where only 13–16% of the patients expressed adverse effects compared to 35% in docetaxel-treated patients [34].

The efficacy and safety of pembrolizumab as a first-line agent in NSCLC gave more promising results than nivolumab against chemotherapy. The ORR with pembrolizumab as a first-line therapy was 44.8%. However, the ORR of the docetaxel-treated group was only 27.8% with 2.2 months as the median time of response to both groups [54]. Studies revealed another potential application of pembrolizumab, i.e., the treatment of recurrent and metastatic urothelial cancer that may include the urethra, the ureter, or the pelvis [55]. The lack of a defined effective therapy for this type of cancer illustrates the importance of pembrolizumab’s role, which is demonstrated in the documented results in comparison to the conventional therapy [55].

The antitumor efficacy of pembrolizumab according to the results of the KEYNOTE-012 study was durable; it revealed a good response rate. About 33 urothelial cancer patients were enrolled in this phase IB study to receive pembrolizumab at a dose of 10 mg/kg every two weeks. The retrieved ORR was 38% among the PD-L1-positive patients and 5% of the pembrolizumab-treated patients displayed grade 3–4 adverse effects [35].

Currently, a phase II study (KEYNOTE-052) is running, in which urothelial cancer patients are enrolled to receive pembrolizumab as a first-line agent [56]. If this study manages to prove the efficacy of pembrolizumab, it will replace cisplatin, the conventional indicated drug for urothelial cancer. The treatment with cisplatin requires a functional kidney which restricts the number of eligible patients [57]. Therefore, pembrolizumab would improve cancer prognosis and broaden the spectrum of patients’ eligibility for the available treatment. 

MM is another FDA approved indication of pembrolizumab as an anticancer agent. In KEYNOTE-002, a phase II clinical trial [38], pembrolizumab demonstrated its powerful role and superiority over chemotherapy in the treatment of melanoma patients. In this trial, 540 patients were allocated to three groups; two groups were assigned to receive either 2 mg/kg or 10 mg/kg of pembrolizumab every three weeks, and the third group of patients were given chemotherapy. The higher administered dose of pembrolizumab (10 mg/kg) resulted in the best progression-free survival (PFS) percent at six months (38%) compared to 34% and 16% in the 2 mg/kg group and in the chemotherapy-treated group, respectively. However, the best tolerance among patients was revealed in the group treated with 2 mg/kg of pembrolizumab, in which only 11% of them showed adverse effects, whereas 14% and 26% of the patients who were given 10 mg/kg of pembrolizumab and chemotherapy, respectively, experienced adverse effects [38].

Furthermore, the efficacy of pembrolizumab in MM patients was demonstrated in another large phase I clinical trial, which enrolled MM patients to receive pembrolizumab at a dose of 2 mg/kg or 10 mg/kg once every two or three weeks [36]. The highest ORR (49%) was achieved in patients treated with 10 mg/kg once every two weeks, which was assessed by RECIST 1.1 through independent central review, and the median PFS was noted to be 31 weeks with a follow-up period exceeding 13 months for all the treated patients [36]. Additional types of tumors were found to be sensitive to the antitumor activity of pembrolizumab as the metastatic and the recurrent nasopharyngeal carcinoma. The KEYNOTE-028 study, a phase IB nonrandomized trial, enrolled 27 pretreated patients to receive pembrolizumab at a dose of 10 mg/kg every two weeks. The ORR among these patients was 25.9% and 15% of them displayed treatment-related adverse events that were classified to be below grade three [37]. The latest study investigating pembrolizumab potency was in triple-negative breast cancer (TNBC) patients. KEYNOTE-012 is a phase IB trial that provided initial evidence on the potential efficacy [39]. In this study, 27 TNBC female patients were enrolled and received 10 mg/kg of pembrolizumab every two weeks; 18.5% was documented as the ORR and no severe toxicities were noted [39]. 

A similar phase IB study (KEYNOTE-028), which was performed in PD-L1-positive ovarian cancer patients, provided promising preliminary results. Among the 26 enrolled patients, one patient achieved a complete response and two patients showed partial response after treatment with 10 mg/kg of pembrolizumab once every two weeks. The best OR was 11.5% and all patients experienced ≥1 adverse events such as fatigue, anemia and decreased appetite [40]. Thus, further investigations may introduce pembrolizumab as an effective and well tolerated agent in the treatment of breast and ovarian cancer. Finally, it was proven that pembrolizumab anticancer activity is not restricted only to solid tumors, but also covers the relapsed or refractory primary mediastinal large B-cell lymphoma (rrPMBCL) which is a challenging tumor to cure. The multi-cohort study, KEYNOTE-013, showed successful outcomes, as the ORR was 41% among the 18 enrolled rrPMBCL patients [41]. Accordingly, we can observe the wide range of anti-cancer activity of pembrolizumab in both solid and hematological malignancies. This finding would also propose pembrolizumab as a powerful treatment option for cancer duplicities in which the patients have more than one type of cancer such as melanoma and B-cell chronic lymphocytic leukemia. Multiple cases on such duplicity were reported owing to immunological defects [58].

Pembrolizumab applications suggest certain similarities to nivolumab-reported studies and findings. Both agents share comparable results in terms of their clinical efficacy in certain tumor types such as in melanoma, in addition to the fact that both were the first FDA approved checkpoint inhibitors for the same indications [59]. However, in NSCLC, nivolumab did not show superiority over chemotherapy as a first-line treatment, unlike pembrolizumab. This variation encouraged many investigators to explore whether such discrepancies are drug-dependent or not by conducting molecular and clinical comparisons. Nivolumab and pembrolizumab were found to be highly related at the molecular level. In addition, in vitro and preclinical experiments did not indicate any drug-dependency. These findings suggest that the reported differences from clinical trials are unlikely to be drug dependent [60].

### 2.3. Pidilizumab

Pidilizumab (CT-011) is an IgG-1 mAb that targets PD-1 protein (Table 1). The efficacy of pidilizumab was found to be due to the induction of T lymphocytes and the anti-cancer activity of natural killer cells [61]. Pidilizumab is particularly potent in different types of hematological malignancies; most of the reported studies and trials exploited its activity against them. Pidilizumab is the first agent to be used clinically for the treatment of diffuse large B-cell lymphoma (DLBCL) [43]. The achieved success in the early phase of clinical trials in DLBCL led the scientists to perform a phase II trial in which patients were given pidilizumab after autologous hematopoietic stem cell transplantation (auto-HSCT) [62]. Among the 66 treated patients, the 16 months OS was 85% and the reported 16 months PFS was 72%. In addition, patients who did not yet possess a measurable malignancy following HSCT, displayed a good response to pidilizumab treatment in 51% of the enrolled cases, where complete remission (CR) and partial remission (PR) were attained in 34% and 27% of the enrolled cases, respectively [42]. Hence, the positive responses and prolongation in PFS periods among post-HSCT Pidilizumab-treated DLBCL patients support the theories behind the pidilizumab antitumor activity in this type of malignancy [42]. Furthermore, a phase I study investigated pidilizumab potency in multiple hematological malignancies. In this study, patients were enrolled with various advanced hematological malignancies including acute myeloid leukemia, multiple myeloma, chronic lymphocytic leukemia, Hodgkin lymphoma and non-Hodgkin lymphoma. The use of pidilizumab displayed a clinical benefit of 33%. The observed durable response was more than 13 months [43].

Besides pidilizumab activity in hematological malignancies, a phase II clinical trial revealed its powerful activity in refractory cases of melanoma [44]. In this study, 103 refractory and previously treated melanoma patients were randomized to receive pidilizumab at two different intravenous doses (1.5 or 6 mg/kg) every two weeks. The OS after one year of treatment was 64.5% with no significant difference between the doses. In conclusion, we can recognize the antitumor activity array of pidilizumab as being more specific to hematologic malignancies; however, this does not eliminate its role in other tumor types as more research is currently ongoing.

### 2.4. AMP-224 and AMP-514

AMP-224 is a fusion protein composed of the programmed death-ligand 2 (PD-L2) extracellular region and the fragment crystallizable (FC) region of the human IgG-2A (Table 1) [63]. Unlike other PD-1 inhibitors, AMP-224 does not induce an immune response by solely blocking the PD-1 receptors; rather, it is suggested that it functions by eliminating exhausted effector cells, i.e., PD-1 highly expressed T cells [63].

Therefore, AMP-224 might exhibit distinct safety and efficacy results as an anticancer agent compared to other PD-1 inhibitors, due to the differences in their biological nature and mechanisms of action. An ongoing study in solid tumors has shown promising tolerance in one patient to a dose of 30 mg/kg. Thus, more crucial outcomes are expected to be revealed by the end of the study [45]. Other reports have displayed the application of AMP-224 in metastatic colorectal cancer, in which adequate peripheral anticancer activity was documented in colorectal cancer patients treated with radiation. However, this positive response upon the administration of AMP-224 might be due to the radiation-induced expression of PD-L1 and this is the current concern that the study is trying to resolve [64].

Among the specific reactions seen with the use of AMP-224 was infusion reactions, which can be explained by the unique nature of this agent [63]. This phenomenon was noted in 69% of patients with advanced solid malignancies across dose cohorts [63]. As a result, the loss of AMP-224 activity and efficacy due to such reaction may hinder its use. 

AMP-514 is another PD-L2 fusion protein that aims to activate immune defense cells by blocking PD-1 [46]. This agent is still under clinical evaluation in multiple running trials; many of them are exploiting its activity, safety and tolerability in solid tumors; there is also a study investigating its potency in combination with other drugs in advanced malignancies [46,47]. Both agents AMP-224 and AMP-514 are in the early stages of clinical trials and their use as anticancer agents is currently under investigation.

## 3. PD-L1 Inhibitors

### 3.1. Atezolizumab

Atezolizumab (MPDL3280A/RG7446) is an IgG-1 humanized antibody that blocks the PD-1/PD-L1 interaction, by targeting the expressed PD-L1 on numerous kinds of malignant cells (Table 2). The blockage of the PD-1/PD-L1 pathway stimulates the immune defense mechanisms against tumors. During the development of this agent, the FC region of IgG-1 was modified in such a way as to limit hyperactivity reactions, including antibody-dependent cell-mediated cytotoxicity and complement-dependent cytotoxicity (CDC), thus decreasing the risks and the side effects associated with atezolizumab use [65]. 

In 2016, atezolizumab became the first agent to gain FDA approval as a second-line treatment of urothelial cancer. This approval was based on the results of a cohort study that enrolled 310 urothelial cancer patients, previously treated with chemotherapy [66]. Atezolizumab was given intravenously at a dose of 1200 mg every three weeks. The ORR indicated the efficacy, which was 14.8%. Nonetheless, 6.5% of the treated patients experienced IRAEs [66]. Furthermore, atezolizumab was an efficient solution to the eligibility obstacle in treating urothelial cancer patients with chemotherapy. In a phase II study, among 119 urothelial cancer patients ineligible to cisplatin, the ORR was 23%, after treatment with atezolizumab as a first-line agent [67].

Numerous trials have proven the efficacy of atezolizumab in NSCLC treatment, which pushed the FDA to approve its use as a second-line treatment for previously treated advanced NSCLC patients who are expressing PD-L1 [79]. In a phase II study, 287 NSCLC patients were randomized to receive either docetaxel or atezolizumab. A median OS of 12.6 months was recorded in the atezolizumab-treated patients compared to 9.7 months in the docetaxel group (*p* = 0.04) [68]. In addition, a phase III study that enrolled 1225 patients with NSCLC, has also proved the superiority of atezolizumab over docetaxel. PD-L1-positive patients treated with atezolizumab presented an ORR (14%) which was better than the ORR (13%) for the docetaxel-treated group [69]. When atezolizumab was given to PD-L1-positive NSCLC patients as a first-line agent, it achieved an ORR of 27% according to the BIRCH trial [80].

Atezolizumab was found to be effective against other types of cancer as well. Durable ORR and promising tolerability were noted in a phase I clinical trial preformed with 45 MM patients. The enrolled patients received atezolizumab every three weeks at a dose of 20 mg/kg. The observed PFS for 24 weeks was 35% and the ORR was 26% [70]. In addition, TNBC was successfully managed by atezolizumab; multiple trials have demonstrated its efficacy, thus promoting atezolizumab to phase III trials. Another related phase I study enrolled 21 TNBC patients to receive atezolizumab at a dose of 1200 mg every three weeks, resulting in a 24-week PFS of 33% [71].

The IMmotion150 study declared interesting findings about the immunotherapy role in kidney cancer treatment. The goal of this study was to explore the efficacy of atezolizumab in local or metastatic renal cancer patients and to compare it to the effectivity of sunitinib, a tyrosine kinase inhibitor. A total of 305 renal cancer patients were randomized into three groups to receive either atezolizumab alone or in combination with an angiogenesis inhibitor agent such as bevacizumab, and the third group was treated with sunitinib [72]. The study did not reveal a major difference between the three groups as the measured median PFS was 6.1, 11.7 and 8.4 months for atezolizumab, combination of atezolizumab and bevacizumab, and sunitinib groups, respectively. In addition, the treatment with atezolizumab in both groups was of good tolerability with no major safety concerns [72]. Thus, based on these promising results, a phase III trial is currently running to compare atezolizumab in combination with bevacizumab against sunitinib [81]. The current research on atezolizumab has the potential to provide powerful evidence to extend its FDA approval for tumors other than urothelial and NSCLC.

### 3.2. Durvalumab

Durvalumab (MEDI4736) is a humanized IgG-1 antibody designed to block the PD-L1. It has been designed in a similar fashion to atezolizumab with modifications to the FC region for minimal immune reactions (Table 2) [82]. Among the studies executed with this agent, some trials are exploring the efficacy of durvalumab in pancreatic cancer treatment. A phase IB/II study aimed to compare the safety and effectiveness of durvalumab and ibrutinib, a tyrosine kinase inhibitor, in pancreatic ductal adenocarcinoma (PDA) [83]. In addition, two phase I studies were carried out to evaluate durvalumab as a monotherapy in PDA [84]. Several more trials explored durvalumab use in PDA, either as a monotherapy or in combination regimens. However, the results of most of these trials are still pending. One of these trials tested durvalumab against NSCLS in 198 patients who received durvalumab (10 mg/kg every two weeks) [73]. The observed ORR among these patients was 14% while about 48% of them displayed adverse effects below grade 3 [73]. In another study, which was performed on multiple types of cancer, durvalumab resulted in an ORR of 25% when administered at a dose of 10 mg/kg every three weeks. Adverse events were reported in 33% of the patients with only 7% classified as grade 3 [74].

Moreover, durvalumab was extensively studied in urothelial cancer. A phase IB study reported its efficacy in advanced urothelial bladder cancer patients with an ORR of 31%. Furthermore, a good safety profile was attained in this trial as the most reported side effects were fatigue and decreased appetite and only 4% of these patients showed grade 3 treatment-related adverse events [75]. These positive findings support the results of other studies and trials; hence, it was not surprising that durvalumab eventually was approved by the FDA as a second-line treatment option for local or advanced urothelial carcinoma [85].

The continued success of this agent encouraged the researchers to explore its efficacy and potential anti-cancer activity in several treatment combinations, aiming to come up with the optimum cure. However, the safety of this agent is still not fully guaranteed as its combination with other agents increased the potential toxicity. For example, an administered combination of durvalumab with a CTLA-4 inhibitor, tremelimumab, resulted in inflammatory myopathy in a 68-year-old NSCLC patient; fortunately, this adverse effect was resolved upon the discontinuation of this regimen [86].

### 3.3. Avelumab

Avelumab (MSB0010718C) is a distinguished IgG-1 antibody that inhibits PD-L1 [87]. It differs from other developed PD-L1 inhibitors by its unique ability to activate the innate immune system and by its dual mode of action, as it can eliminate malignancies by both the effector T cells and antibody-dependent cell-mediated cytotoxicity (ADCC) (Table 2). Despite the fact that these mAbs are either designed or engineered to eliminate ADCC, ADCC has, however, been implicated as an important mechanism in several highly effective mAb-mediated cancer therapies [87]. Avelumab efficacy is currently under investigation across 16 types of tumors by a series of large international clinical trials called JAVELIN clinical trials [87]. Among these trials is the phase I multi-cohort JAVELIN solid tumor study, which is described as one of the largest studies to date, as it enrolled more than 1700 patients. It focused on examining the avelumab efficacy in 12 different types of tumors, including breast cancer, NSCLC, skin cancer (melanoma), ovarian cancer, renal cancer, adrenocortical cancer, urothelial cancer, gastric cancer, and head and neck cancer [76]. In this study, avelumab was given every two weeks in a dose escalation manner to achieve 10 mg/kg. Positive initial responses were noticed among NSCLC, gastric cancer, advanced metastatic breast cancer, metastatic Merkel cell carcinoma (mMCC) and urothelial carcinoma. The available safety data of this study indicate that across the different tumor types, avelumab was well tolerated by the patients. In addition, the observed adverse events were mostly below grade 3, with very minimal responses above it [76].

The JAVELIN solid tumor studies include a phase IB trial in metastatic urothelial cancer (MUC), which showed impressive outcomes. Avelumab was administered as a second-line treatment to 44 MUC patients and resulted in an ORR of 40%, in addition to 12 weeks PFS of 70%. Similar to durvalumab, these positive findings in the management of MUC by avelumab pushed the FDA to accelerate its approval as a second-line treatment [77].

The most exciting fact revealed about avelumab is its astonishing efficacy in a very aggressive immunogenic skin cancer type called mMCC. In March 2017, avelumab succeeded in obtaining FDA approval as an anti-cancer agent to cure mMCC [88]. The JAVELIN Merkel 200 phase II study presented evidence supporting the success of avelumab in mMCC treatment in which out of the 88 metastatic MCC patients receiving avelumab, 9.6% had complete remission. In addition, the ORR was 31.8% with six months PFS of 40% [78].

Beyond any doubt, avelumab is one of the unique PD-L1 inhibitors that tapered difficult malignancies such as mMMC, and it gave hope to mMMC patients after the absence of approved treatments for a long period. Most of the research on avelumab is still in progress, just like other PD-1/PD-Ll agents; the complete picture of this promising agent is yet to be presented as it might be the answer to many more pressing problems in the oncology field.

## 4. Conclusions

Targeting PD-1/PD-L1 axes in an attempt to eradicate malignancies, via the activation of the immune system, demonstrated great success in numerous types of tumors. To date, the cumulative research efforts lead to the use of PD-1/PD-L1 inhibitors as official FDA approved agents in many tumors such as MUC, NSCLC, MM and MCC. In fact, the clinical field is very active nowadays, running trials to further evaluate the safety and efficacy of these agents [14]. It is noteworthy to mention that almost all the examined agents share positive results in similar types of malignancies. Yet, some of them demonstrated superiority such as the excellence of avelumab in the treatment of mMCC and the recognized potency of pidilizumab in hematological malignancies.

When it comes to comparing PD-1/PD-L1 inhibitors to classical chemotherapeutic agents, it is clearly observed that some of them can be considered as equals. This was revealed in multiple reported studies of PD-1/PD-L1 inhibitors such as nivolumab and pembrolizumab, which presented similar, and in some cases even higher, responses regarding the potency in addition to being safer in different malignancies compared to standard chemotherapeutic agents [26,53]. However, to date, these impressive reports have not been able to eliminate the role of other chemotherapeutic agents; nevertheless, a huge number of PD-1/PD-L1 agents are still under development. Future studies might achieve the auspicious aims. 

In addition, the use of PD-1/PD-L1 agents is not as well established as the detailed defined use of chemotherapy. In fact, many trials are currently running with the aim of identifying the safest dosage regimens and establishing the most potent and proper combination of the PD-1/PD-L1 inhibitors with chemotherapy or any other known treatment for cancer. These combinations could be a therapeutic option with outstanding results, but the low safety might be the limiting factor, which is an obstacle that many trials are trying to solve. Another drawback against the application of these antibodies is the incidence of IRAEs, which can lead to fatal events on rare occasions and so cannot guarantee the safety of the patients.

The low expression of PD-L1 in cancer cells is another limitation for the use of PD-1/PD-L1 inhibitors due to the direct correlation between the expression level of PD-L1 and therapeutic response. Therefore, the lower the level of PD-L1, the less the expected therapeutic outcome. Many studies have shown that the patient’s response to these agents depends on the level of the PD-L1 expression. This fact was illustrated by collective reports which showed that out of 1400 essayed patients, response was noted in 45% of the PD-L1-positive patients, while in the PD-L1-negative patients, only 15% showed a similar response. Hence, this offers certain selectivity regarding the responsiveness of patients to the use of PD-1/PD-L1 inhibitors [89,90].

Since these agents target immunity, they may result in abnormal immune reactions and generate toxicity that affects the skin, gut, lung, liver and other tissues [91]. Among the IRAEs are the documented cases of ILD following treatment with nivolumab and the neurological complications documented with the administration of both nivolumab and pembrolizumab [91]. Therefore, it is evident that more investment is needed in the area of PD-1/PD-L1 inhibitors to come up with definite figures and numbers regarding the frequency and incidents of these IRAEs. More efforts should also be made to define the most proper protocols for managing these complications without interfering with the anticancer effect.

In conclusion, based on the reported basic and translational studies, the continuous research and development of PD-1/PD-L1 inhibitors has fostered breakthrough findings in the treatment of challenging tumors. Considering the safety of these agents, PD-1/PD-L1 inhibitors are generally better tolerated than classical chemotherapeutic agents. Nevertheless, more preclinical and clinical investigations are required to elucidate the key mechanisms and predictive biomarkers of the efficacy and safety of these agents and to limit the risk of IRAEs.

## Figures and Tables

**Table 1:** PD-1- inhibitors.

Drug	Phase	Indication (Case)	Number of Patients	Median OS	ORR (%)	PFS	Control Drug	NCT Number	Ref.
**Nivolumab**	**III**	**B-RAF negative MM**	**418**	**-**	**40**	**5.1 m**	**Dacarbazine**	**NCT01721772**	**[27]**
	**I/II**	**Melanoma pretreated**	**107**	**17 m**	**31**	**3.7 m**	**-**	**NCT00730639**	**[23]**
	**III**	**Advanced NSCLC**	**272**	**9.2 m**	**20**	**3.5 m**	**Docetaxel**	**NCT01642004**	**[28]**
	**III**	**Advanced NSCLC**	**582**	**12.2 m**	**19**	**2.3 m**	**Docetaxel**	**NCT01673867**	**[28,29]**
	**I**	**NSCLC**	**52**	**19.4 m**	**28**	**3.6 m**	**Docetaxel**	**NCT01454102**	**[30]**
	**III**	**GEJC**	**493**	**5.32 m**	**11.2**	**1.61 m**	**Placebo**	**NCT02267343**	**[31]**
	**I/II**	**Advanced and metastatic GEJC**	**59**	**6.8 m**	**18**	**-**	**-**	**NCT01928394**	**[32]**
	**III**	**Head and neck cancer**	**361**	**7.5 m**	**-**	**2 m**	**Methotrexate/Docetaxel/Cetuximab**	**NCT02105636**	**[25]**
	**II**	**Ovarian cancer**	**20**	**20 m**	**-**	**3.5 m**	**-**	**-**	**[33]**
**Pembrolizumab**	**II/III**	**NSCLC**	**1034**	**12.7 m**	**44.8**	**4 m**	**Docetaxel**	**NCT01905657**	**[34]**
	**IB**	**Urothelial cancer**	**33**	**-**	**38**	**-**	**-**	**NCT02335424**	**[35]**
	**II**	**MM**	**150**	**-**	**-**	**31 wk**	**-**	**-**	**[36]**
	**IB**	**Metastatic nasopharyngeal carcinoma**	**27**	**-**	**25.9**	**-**	**-**	**NCT02054806**	**[37]**
	**I**	**MM**	**135**	**-**	**41**	**38% (10 mg/kg)**	**-**	**NCT1295827**	**[38]**
	**IB**	**TNBC**	**27**	**-**	**18.5**	**-**	**-**	**NCT02447003**	**[39]**
	**IB**	**Ovarian cancer**	**26**	**-**	**-**	**-**	**-**	**NCT02054806**	**[40]**
	**IB**	**rrPMBCL**	**18**	**-**	**41**	**-**	**-**	**NCT01953692.**	**[41]**
**Pizilizumab**	**II**	**DLBCL**	**66**	**-**	**72**	**72%**		**NCT00532259**	**[42]**
	**I**	**Hematological malignancies**	**17**	**-**	**-**	**-**	**-**	**-**	**[43]**
	**II**	**Melanoma**	**103**	**-**	**5.9**	**2.8 m**	**-**	**NCT01435369**	**[44]**
**AMP-224**	**I**	**Solid tumors**	**6**	**-**	**-**	**-**	**-**	**-**	**[45]**
**AMP-514**	**I**	**Advanced malignancies**	**150**	**-**	**-**	**-**	**-**	**NCT02118337**	**[46,47]**

**NCT**, National clinical trial; **PFS**, Progression free survival; **OS**, Overall survival; **ORR**, Objective response rate; **MM**, Metastatic melanoma; **NSCLC**, Non-small cell lung cancer; **GEJC**, Gastroesophageal junction cancer; **TNBC**, Triple negative breast cancer; **rrPMBCL**, Relapsed or refractory primary mediastinal large B-cell lymphoma; **DLBCL**, Diffuse large B-cell lymphoma.

**Table 2:** PD-L1 inhibitors.

Drug	Clinical Phase	Indication (Case)	Number of Patients	OS	ORR (%)	PFS	Control Drug	NCT Number	Ref.
**Atezolizumab**	**-**	**Urothelial cancer**	**310**	**-**	**14.8**	**-**	**-**	**NCT02108652**	**[66]**
	**II**	**Urothelial cancer**	**119**	**15.9 m**	**23**	**2.7 m**	**-**	**NCT02108652**	**[67]**
	**II**	**NSCLC**	**287**	**12.6 m**	**-**	**-**	**Docetaxel**	**NCT01903993**	**[68]**
	**III**	**NSCLC**	**1225**	**13.8 m**	**13**	**2.8 m**	**Docetaxel**	**NCT02008227**	**[69]**
	**I**	**MM**	**45**	**-**	**26**	**35%**	**-**	**NCT01375842**	**[70]**
	**I**	**TNBC**	**21**	**-**	**24**	**-**	**-**	**NCT01375842**	**[71]**
	**II**	**mRCC**	**305**	**-**	**-**	**6.1 m**	**Sunitinib**	**NCT01984242**	**[72]**
**Durvalumab**	**I/II**	**NSCLC**	**198**	**-**	**14**	**-**	**-**	**NCT01693562**	**[73]**
	**I/II**	**Advanced solid tumors**	**151**	**-**	**-**	**-**	**-**	**NCT01693562**	**[74]**
	**IB**	**Urothelial bladder cancer**	**61**	**-**	**31**	**-**	**-**	**NCT01693562**	**[75]**
**Avelumab**	**I**	**Solid tumors**	**>1700**	**-**	**-**	**-**	**-**	**NCT01772004**	**[76]**
	**IB**	**MUC**	**44**	**-**	**40**	**70%**	**-**	**NCT01772004**	**[77]**
	**II**	**mMCC**	**88**	**11.3 m**	**31.8**	**2.7 m**	**-**	**NCT02155647**	**[78]**

**NCT**, National clinical trial; **PFS**, Progression free survival; **OS**, Overall survival; **ORR**, Objective response rate; **NSCLC**, Non-small cell lung cancer; **MM**, Metastatic melanoma; **TNBC**, Triple negative breast cancer; **mRCC**, Metastatic renal cell carcinoma; **MUC**, Metastatic urothelial cancer; **mMCC**, Metastatic merkel cell carcinoma.
